# Proactive integrated virtual healthcare resource use in primary care

**DOI:** 10.1186/s12913-021-06783-9

**Published:** 2021-08-12

**Authors:** Jolie N. Haun, Bridget A. Cotner, Christine Melillo, Vanessa Panaite, William Messina, Shilpa Patel-Teague, Brian Zilka

**Affiliations:** 1 Research and Development Service, James A. Haley VA Hospital and Clinics, 8900 Grand Oak Circle (151R), Tampa, FL 33637-1022 USA; 2 Department of Community & Family Health, College of Public Health, University of South Florida, Tampa, FL USA; 3 Department of Anthropology, University of South Florida, Tampa, FL USA; 4 Department of Psychology, University of South Florida, Tampa, FL USA; 5 James A. Haley Veterans Hospital, Tampa, FL USA; 6 Veterans Integrated Service Network 8 Network Office, St Petersburg, FL USA

**Keywords:** Veteran, Technology, Clinical decision support, Patient engagement, Primary care, Virtual healthcare resources

## Abstract

**Background:**

Proactive integrated virtual healthcare resource (VHR) use can improve efficiency, maximize resource capacity for delivering optimal coordinated care and improve patient outcomes. Proactive integrated VHR use is vital for delivering high quality care. Our objectives were to identify proactive integrated VHR use among primary care teams, best practices and targeted implementation strategies to promote proactive integrated VHR use.

**Methods:**

This is a mixed-method descriptive study. We employed a community-based participatory approach to collect data and the Consolidated Framework for Implementation Research to analyze and contextualize findings. A cross-sectional sample of primary care team members (*n* = 65) from a Department of Veterans Affairs medical center participated in focus groups, follow-up interviews (*n* = 16), and respond to self-report surveys. Operational subject matter experts (*n* = 15) participated in informant interviews.

**Results:**

Survey data described current use and factors that influenced singular VHR use and were convergent with qualitative findings. Focus group and interview data described no evidence of proactive integrated VHR use. Differences and similarities were identified between both utilization groups, such as facilitators and barriers, recommendations, patient education and preferred implementation strategies. All groups reported issues around VHR availability knowledge and access and functionality. Participants identified the need for best practices that are specific to care tasks and performance measures. Expert informant interviews identified a list of VHR tools that could be proactively integrated across the healthcare continuum.

**Conclusions:**

Health systems are leveraging technologies to proactively integrate VHR to maximize information exchange, clinical decision support and patient engagement. VHR is critical during global pandemics, such as COVID-19, to maintain access to care coordination and delivery while abiding by public health recommendations. Though recent requirements for reducing contact create an intrinsic motivation, cultural change through education and best practices of proactive integrated use across the healthcare continuum is needed to create a culture of VHR super users.

**Supplementary Information:**

The online version contains supplementary material available at 10.1186/s12913-021-06783-9.

## Background

Virtual healthcare resources (VHR), also known as eHealth and telehealth [1], are central to strategic priorities for ensuring receipt of timely, integrated, patient-centered care. VHR consist of many resources including patient health portals, mobile technology, electronic health records, and telehealth including video, audio, instant and secure messaging [1]. Healthcare systems are taking strategic steps to promote VHR use to support access, care coordination and delivery needs through an extensive network of resources, to improve patient outcomes and promote efficient system utilization [2]. Healthcare systems have established VHR as an option for care delivery. In light of COVID-19, most systems are now relying on VHR to coordinate and deliver high quality care *while* reducing contact [3]. The imminent need for proactive integrated VHR use in healthcare systems warrants investigation.

Proactive integrated VHR use is defined as a self-initiated approach to coordinated use of applicable VHR systems for the purposes of coordinating and delivering timely high-quality patient-centered care [4]. Fragmented systems can frustrate primary care teams, and reduce patients’ adoption of VHR and satisfaction with care [5, 6]. VHR is most effective when tools are used in an integrated manner which improves workflow efficiency [7]. Provider promotion and proactive reinforcement are key factors in patient adoption and sustained use of VHR [5, 6]. Understanding best practices of VHR proactive integrated use can optimize VHR implementation efforts.

Primary care typically includes a physician, nurse, clinical and clerical associates [8]. This primary care team can extend to staff members from specialty areas of service, such as pharmacy, nutrition, social work and psychology. Since implementation of primary care teams in the Department of Veterans Affairs (VA), use of VHR has increased, yet little is known about the integrated use of VHR. Currently there are no established best practices to guide clinical practice [9]. The aims of this research were to: (1) examine primary care use and perceptions of VHR among high- and low- utilization groups; (2) determine the level of integration of VHR in primary care, and identify best practices; and (3) identify implementation of strategies to promote primary care use and integration of VHR across the health care continuum [10].

### Theoretical context

A community-based participatory research (CBPR) approach guided data collection and the Consolidated Framework for Implementation Research (CFIR) [11] informed analyses. This study was developed using CBPR [12]. During proposal development team members actively developed collaborative partnerships with primary care team members. This included meetings, resource sharing, networking and open discussions of needs and barriers. The study team intentionally hired clinicians with long term relationships with primary care teams and a shared language to interpret communication as needed. This fledgling group helped to further define the study problem in language that all understood. Partnerships were documented through study documents, meeting minutes and regulatory forms. Given the duration of this study and partnerships, open discussions, feedback and data interpretation were common in weekly meetings. Community partners were involved in all dissemination activities including development of ideas, presentations, video production and manuscripts. Primary care team members (i.e. participants) were actively involved in development of study protocols, data collection, and co-creation and implementation of the intervention [13]. CFIR constructs were used to guide data analysis, document primary care team VHR use, and strengthen the research and applicability of findings [14] to broader implementation efforts.

## Methods

This paper reports findings from an Institutional Review Board approved concurrent mixed-method implementation study [10].

### Setting

This study was conducted within primary care at one VA medical center, located in the southeastern United States. This VA is comprised of six sites: main hospital, primary care annex, and four community-based outpatient clinics.

### Participants

A complete description of participant selection and recruitment is published [10]. In short, using outgoing Secure Messages as a proxy for VHR use, primary care teams were ranked “high” or “low” utilization. Rankings were confirmed by clinical experts following a CBPR approach. Active and passive methods were used to recruit primary care team members (*N* = 267) [15]. Snowball sampling identified extended primary care team members (e.g., pharmacy, social work). Expert informants (*n* = 15), consisting of operational partners and local leaders, were purposively recruited based on their knowledge of VHR systems. Potential participants who did not participate cited reasons such as lack of time or inappropriate fit to study aims.

### Sample

Of the 57 existing teams, 19 were purposively identified based on VHR utilization (10 high [*n* = 10]; low [*n* = 9]). Twenty-one clinic-held focus groups were conducted: 19 high and low groups and 2 extended primary care team focus groups. Of the 267 primary care team members 65 provided written consent to participate. Approximately four primary care team members (i.e. physician, nurse, clinical and clerical associate) participated in each focus group. Following a CBPR approach, we approached focus group participants who provided insightful information to participate in a follow-up interview to provide additional information regarding integration of VHR among other topics. Follow-up interviews were conducted with focus group participants (*n* = 16) at the place of their choice (e.g. clinic). Expert informants (*n* = 15) participated in an individual telephone interview. Only participants and researchers were present for data collection activities.

### Data sources

Data collection measures (survey, focus group discussion and interview guides) were developed for this study and have been previously piloted, described and published (6). The demographic questionnaire (Additional File 1) included 16 items and the CFIR-informed self-report survey (Additional File 2) contained 18 items. A CFIR-informed focus group guide (Additional File 3) included a list of implementation strategies [16]. Participants identified preferred VHR implementation strategies. A semi-structured interview guide (Additional File 4) was used for follow-up interviews and expert informants.

### Data collection procedures

**Fig. 1:**
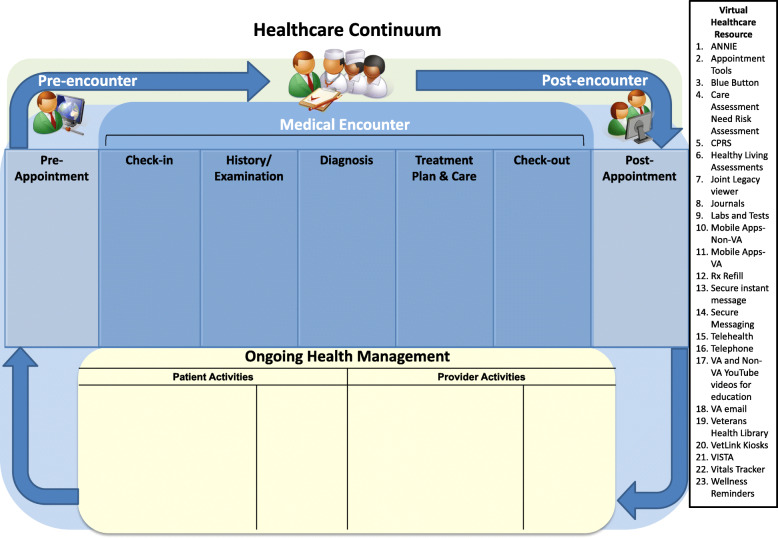
Healthcare continuum and relevant virtual healthcare resources

Prior to data collection, research staff met with all sites to introduce individual researchers (e.g., name, educational background), provide information about the study and explicitly develop rapport. CM had existing professional relationships with several primary care team members. Study demographic and VHR use survey data were collected August through November 2018 and managed using REDCap electronic data capture tools hosted at Veterans Affairs [17, 18]. REDCap (Research Electronic Data Capture) is a secure, web-based software platform designed to support data capture for research studies, providing 1) an intuitive interface for validated data capture; 2) audit trails for tracking data manipulation and export procedures; 3) automated export procedures for seamless data downloads to common statistical packages; and 4) procedures for data integration and interoperability with external sources. Qualitative data collection was conducted by study staff including BC and CM. All interviews and focus groups were audio recorded with permission. December through February 2018 fifty-five minute in-person, clinic-based focus groups were conducted to identify proactive integrated VHR use, best practices and targeted implementation strategies; 30-min follow-up interviews were conducted for clarification and individual perspective. March through May 2019, 30-min expert informant interviews were conducted via telephone to elicit a future vision for an integrated VHR system. Interviewers electronically shared a graphic representation of the healthcare continuum with expert informants (Fig. 1). Expert informants were asked to modify this continuum based on their knowledge of organizational capacity, clinical needs, and patient preferences.

### Data analysis

Descriptive statistics were used by VP to analyze the demographic questionnaire to describe the overall sample and high and low utilization group characteristics. Group means and standard deviations (SD) were also computed for each subscale described in Table 3 by averaging responses across items and across responders; lower scores signified more positive perceptions. Given the size of our samples (High utilization group [*n* = 23] and Low utilization group [*n* = 29]) comparative analyses (t-test and chi-square) of survey data were supplemented by effect size calculations. We calculated Cohen’s d and h effect sizes and 95% confidence intervals [19], commonly used to evaluate differences between two independent means and two independent proportions, respectively. Analyses were performed with SPSS Version 25 (*SPSS Statistics for Windows*, n.d.) to identify group differences between participants associated with high and low utilization user groups. Across all variables, 42.9% had no missing data, 28.6% had < 5 participants with missing data (< 10%), 25.9% had < 10 participants with missing data (< 20%). The remaining 3 variables had 11–18 participants with missing data. Overall, 384 (6.59%) of 5824 values were missing completely at random. Therefore, the default listwise deletion was deemed acceptable.

Qualitative data collection and analysis were conducted by BC and CM and research staff concurrently. Insights from data analyses were used to guide subsequent data collection. A rapid analysis process [20] was used to analyze interview data. Data were managed within Microsoft Excel. Content analysis was conducted by four research team members who read d participant comments, coded the data deductively by CFIR constructs, and merged similar codes to identify themes [21–24]. Data were stratified by high and low utilization groups and compared to one another. Matrix analysis was used to analyze across-thematic categories [25]. Clinical team members and expert informants verified findings and provided clarification.

## Results

### Survey findings: utilization patterns and beliefs about VHR

Participants represented all roles on a primary care team and were mainly white females over 46 years old (Table 1). Survey data described participants’ VHR use, perceptions of patient’s preferred methods of communication, and promotion of VHR with their patients.

**Table 1:** Primary Care Participant Characteristics

	Total (N = 65)	HIGH (***n*** = 23)	LOW (***n*** = 29)	Extended Primary Care Team (***n*** = 13)	Follow-up Interview (***n*** = 16)
**Characteristic**	N (%)	n (%)	n (%)	n (%)	n (%)
**Role**					
Provider	12 (18)	6 (50)	6 (50)	0 (0)	3 (25)
Nurse	14 (22)	5 (36)	9 (64)	0 (0)	7 (50)
Clinical Associate	14 (22)	6 (43)	8 (57)	0 (0)	4 (29)
Clerical Associate	12 (18)	6 (50)	6 (50)	0 (0)	2 (17)
Other (Pharmacists, Psychologists, Dietician, Social Worker)	13 (20)	0 (0)	0 (0)	13 (100)	0 (0)
**Race** ^**a**^					
White, Caucasian	46 (69)	13 (57)	22 (76)	11 (92)	12 (75)
Black, African American	10 (15)	6 (26)	3 (10)	1 (8)	1 (6)
Other ^b^	11 (17)	4 (17)	4 (14)	3 (23)	3 (19)
**Ethnicity**					
Hispanic	8 (13)	3 (13)	1 (3)	4 (33)	0 (0)
Not Hispanic	44 (75)	13 (57)	24 (83)	7 (58)	10 (63)
Decline to Respond	7 (12)	7 (30)	4 (14)	1 (8)	6 (38)
**Sex**					
Female	48 (75)	19 (83)	21 (72)	8 (67)	14 (88)
**Age**					
25–35	14 (24)	3 (14)	2 (10)	7 (64)	0 (0)
36–45	10 (17)	3 (14)	6 (29)	3 (27)	3 (23)
46–55	14 (24)	8 (38)	8 (38)	1 (9)	6 (46)
56–60	15 (26)	4 (19)	2 (10)	0 (0)	2 (15)
61 +	5 (9)	2 (10)	3 (14)	0 (0)	2 (15)

^a^ Note: participants selected multiple racial categories, so the percentages add up to more than 100%

^b^ Note: Other category includes Asian (Chinese, Filipino, Japanese, Korean etc.), Native Hawaiian or other Pacific Islander, American Indian or Alaskan Native, Unknown and Other

Most participants reported using and promoting My Health***e***Vet, Secure Messaging, and VetLink Kiosks with patients, across low and high user groups. Although telehealth use was reported by nearly 50% of all providers, mobile apps use was the lowest used resource across participant groups (Table 2). More than two thirds in both utilization groups reported using VHR tools daily (Low group = 69% yes daily; High group = 78.3% yes daily; ^2^ = 0.58, *p* = 0.57, h (95%CI) = 0.21 (0.14,0.29)) and over two thirds of the providers across groups have been using VHR for over 3–5 years and more (Low group = 65.5% yes 3–5+ years; High group = 65.2% yes 3–5+ years; ^2^ = 0.65, *p* = 0.52, h (95%CI) = 0.01 (− 0.07,0.08).

**Table 2:** Use and promotion of virtual healthcare resources (VHR) among providers from high versus low utilization groups

	LOW (N = 29)	HIGH (n = 23)	X^***2***^	***p***	***h (95%CI)***
**Provider’s VHR use (% yes)**					
My Health***e***Vet	79.3	73.9	0.21	0.65	0.13 (0.05,0.20)
Secure Messaging	86.2	78.3	0.57	0.45	0.21 (0.13,0.28)
Telehealth	48.3	43.5	0.12	0.73	0.10 (0.02,0.17)
VetLink Kiosks	51.7	69.6	1.70	0.19	0.37 (0.29,0.44)
Mobile Apps	10.3	13.0	0.09	0.76	0.08 (0.01,0.16)
**Patients’ preferred methods of communication (% yes)**					
Telephone	86.2	95.7	1.32	0.25	0.34 (0.27,0.42)
Face to face*	79.3	95.7	2.94	0.09	0.53 (0.45,0.60)
My Health***e***Vet	34.5	43.5	0.44	0.51	0.18 (0.11,0.26)
Secure Messaging*	65.5	87.0	3.14	0.08	0.52 (0.44,0.59)
Telehealth	13.8	8.7	0.33	0.57	0.16 (0.09,0.24)
VetLink Kiosks	20.7	13.0	0.52	0.47	0.21 (0.13,0.28)
Mobile Apps	3.4	8.7	0.65	0.42	0.23 (0.15,0.30)
**Providers’ promotion of patients’ use of VHR (% yes)**					
My Health***e***Vet	86.2	87.0	0.01	0.94	0.02 (−0.05,0.09)
Secure Messaging	93.1	95.7	0.15	0.70	0.11 (0.04,0.18)
Telehealth	48.3	39.1	0.44	0.51	0.19 (0.12,0.26)
VetLink Kiosks	58.6	47.8	0.60	0.44	0.22 (0.15,0.29)
Mobile App	10.3	13.0	0.09	0.76	0.08 (0.01,0.15)
**Promotion of patients’ use of VHR on behalf of providers (% yes)**					
My Health***e***Vet	3.7	5.3	0.21	0.90	0.08 (0.01,0.15)
Secure Messaging	0.0	4.8	3.63	0.16	0.44 (0.37,0.51)
Telehealth	3.6	5.0	0.33	0.95	0.07 (−0.01,0.14)
VetLink Kiosks	16.7	5.3	1.91	0.39	0.38 (0.31,0.45)
Mobile Apps	12.5	5.6	1.37	0.71	0.24 (0.17,0.31)
**% Patients with whom use/promote VHR (% responded 50–100%)**					
My Health***e***Vet	55.6	70.0	1.01	0.31	0.30 (0.23,0.37)
Secure Messaging	63.0	68.4	0.15	0.70	0.11 (0.04,0.18)
Telehealth	20.8	31.3	0.56	0.46	0.24 (0.17,0.31)
VetLink Kiosks	56.5	57.9	0.01	0.93	0.03 (−0.04,0.10)
Mobile Apps	11.8	26.7	1.16	0.28	0.38 (0.31,0.45)

* *p* < .10

Providers from both user groups perceived patients’ preferred methods of communication to be primarily telephone, face to face, and Secure Messaging, followed by My Health***e***Vet tools (below 50%), with telehealth, VetLink Kiosks, and apps reported as the least preferred among patients (Table 2). Although none of the group differences in reported patient’s preferences reached traditional statistical significance threshold, high utilization providers reported more elevated face to face and Secure Messaging preferences among their patients relative to low utilizing providers. These differences did not reach significance levels (*p* > .05) however the observed medium effect sizes (h > .50) suggest that the analysis was under powered to detect even a medium effect. Survey data suggest promotion of Secure Messaging followed by My Health***e***Vet was high among all providers, with more than 85% of providers from both high and low utilizing groups endorsing these tools. Even promotion of telehealth and VetLink Kiosk use was relatively high across providers (Table 2).

Participants perceived relative advantage (i.e. improved care delivery, preference over traditional tools), observability (i.e., improved clinical workflow and patient outcomes), and compatibility (i.e. how well VHR fits with primary care team values and workflow systems) for the use of My Health**e**Vet and Secure Messaging; however, data suggests provider perceptions of telehealth and VetLink Kiosks were more mixed in terms of their perceived relative advantage in patient care and workflow (Table 3). Survey data captured perceptions of complexity (i.e. perceived difficulty of implementing use of VHR) through two subscales: Compatibility and Complexity. Responses suggest while providers from low use groups consistently reported comfort with utilization of My Health***e***Vet and Secure Messaging, providers from high use groups reported more variability in comfort with both My Health***e***Vet and Secure Messaging. Although differences between providers from high and low use groups did not reach traditional statistical significance thresholds (i.e., *p* < .05), effect sizes crossed the medium threshold for My Health***e***Vet and Secure Messaging suggesting meaningful differences between the groups on perceptions about compatibility of VHR use. Next, the complexity subscale showed that, on average, providers reported ease with integrating all VHR and that they would benefit from education on how to access tools with patients (Table 3). Finally, the context and facilitation (i.e., use reflects role responsibilities, reinforced by workplace) survey subscale highlighted My Health***e***Vet and Secure Messaging use across both groups; use was reinforced in the workplace; and both were most often used and recommended for patient use. Again, reports varied related to telehealth, VetLink Kiosks, and apps (Table 3).

**Table 3:** Perceptions about virtual healthcare resources (VHR) among providers from high versus low use teamlets

	LOW (N = 29)	HIGH (n = 23)	***t***	***p***	***d (95%CI)***
**Relative advantage** (improved care delivery, preference over traditional tools) **(M, SD)**					
My Health***e***Vet	1.81 (.76)	2.03 (1.14)	−0.79	0.43	0.23 (−0.32,0.78)
Secure Messaging	1.72 (.84)	1.93 (.90)	−0.83	0.41	0.24 (−0.31,0.79)
Telehealth	2.30 (1.20)	2.60 (1.69)	−0.71	0.48	0.20 (−0.34,0.75)
VetLink Kiosks	2.72 (1.41)	2.60 (1.53)	0.29	0.77	0.08 (−0.47,0.63)
Mobile Apps	3.27 (1.67)	3.11 (1.68)	0.33	0.75	0.10 (−0.45,0.64)
**Observability** (improved clinical workflow and patient outcomes) **(M, SD)**					
My Health***e***Vet	1.81 (.71)	2.08 (1.24)	−0.95	0.35	0.27 (−0.28,0.82)
Secure Messaging	1.69 (.67)	1.93 (.73)	−1.16	0.25	0.34 (−0.21,0.89)
Telehealth	2.22 (1.29)	2.83 (1.74)	−1.39	0.17	0.40 (−0.15,0.95)
VetLink Kiosks	2.52 (1.15)	2.90 (1.74)	−0.86	0.40	0.26 (−0.29,0.81)
Mobile Apps	3.23 (1.61)	3.13 (1.71)	0.22	0.83	0.06 (−0.49,0.61)
**Compatibility** (comfort with VHR use to communicate and deliver care) **(M, SD)**					
My Health***e***Vet*	1.71 (.81)	2.30 (1.22)	−2.00	0.05	0.57 (0.01,1.13)
Secure Messaging*	1.55 (.74)	2.05 (1.02)	−2.00	0.05	0.56 (0.003,1.12)
Telehealth	2.78 (1.53)	3.20 (2.07)	−0.77	0.45	0.23 (−0.32,0.78)
VetLink Kiosks	2.52 (1.35)	3.24 (2.10)	−1.38	0.18	0.41 (−0.14,0.96)
Mobile Apps	3.17 (1.47)	3.68 (1.89)	−0.98	0.33	0.30 (−0.25,0.85)
**Complexity** (education is needed on access/use, integration ease in patient care delivery) **(M, SD)**					
My Health***e***Vet	2.00 (.73)	1.98 (1.13)	0.09	0.93	0.02 (−0.53,0.57)
Secure Messaging	2.02 (.70)	1.81 (.70)	1.04	0.31	0.30 (−0.25,0.85)
Telehealth	2.47 (1.22)	2.52 (1.47)	−0.15	0.88	0.04 (−0.51,0.58)
VetLink Kiosks	2.54 (1.19)	2.67 (1.52)	−0.33	0.74	0.10 (−0.45,0.64)
Mobile Apps	2.80 (1.59)	2.98 (1.45)	−0.39	0.70	0.12 (−0.43,0.67)
**Context and facilitation** (use reflects role responsibilities, reinforced by workplace) **(M, SD)**					
My Health***e***Vet	1.92 (1.04)	2.11 (1.23)	−0.55	0.59	0.17 (−0.38,0.72)
Secure Messaging	1.83 (1.01)	1.80 (.65)	0.11	0.92	0.04 (−0.51,0.58)
Telehealth	2.44 (1.33)	2.70 (1.57)	−0.62	0.54	0.18 (−0.37,0.73)
VetLink Kiosks	2.58 (1.31)	2.52 (1.52)	0.13	0.90	0.04 (−0.51,0.59)
Mobile Apps	3.25 (1.45)	3.14 (1.60)	0.23	0.82	0.07 (−0.48,0.62)

* *p* < .10

### Focus group and follow-up interview findings

Focus group and follow-up interview findings for primary care and extended primary care members were consistent and thus combined for results presentation. Of key interest was the differences between high and low user groups, surprisingly, differences were minimal, but some did emerge, such that: (1) low user groups tended to report using a more structured process for VHR patient education than high user groups; (2) high user groups identified the need for properly functioning, integrated and accessible equipment, while low user groups recommended policy/system changes such as work load credit, workflow fit, decreased workload, safety nets for communication with patients and dedicated staff to educate patients; (3) high user groups identified factors which inhibit VHR use, such as lack of accessibility and function, while low user groups focused on factors such as individual patient needs and preferences, and staff lack of knowledge about VHR; (4) high user groups identified ways to overcome barriers, such as mandates and awareness of VHR benefits (e.g. less trips to clinic, less phone calls, redistributed work), while low user groups identified process structures (e.g. triage processes, follow-up processes) to overcome barriers; and (5) though both groups identified education and training as preferred implementation strategies, low user groups identified more implementation strategies to support VHR use (Table 4).

**Table 4:** High and Low Utilization Groups’ Most Useful Recommended Implementation Strategies

High Utilization Groups’ Recommended Implementation Strategies	%
Develop educational materials	40
Change record systems	40
Centralize technical assistance	33
Assess for readiness and identify barriers and facilitators	33
Low Utilization Groups’ Most Useful Implementation Strategies	
Centralize technical assistance	61
Conduct ongoing training	61
Conduct educational outreach visits	61
Capture and share local knowledge	57
Change physical structure and equipment	57
Conduct educational meetings	57
Build a coalition	57
Distribute educational materials	57
Develop educational materials	43
Involve patients/consumers and family members	43
Assess for readiness and identify barriers and facilitators	39
Prepare patients/consumers to be active participants	39
Access new funding	39
Conduct local consensus discussions	39
Audit and provide feedback	39
Intervene with patients/consumers to enhance uptake and adherence	35
Make training dynamic	35
Obtain and use patients/consumers and family feedback	35
Conduct local needs assessment	30
Develop academic partnerships	30

Qualitative analysis also identified factors that emerged relevant to VHR use among all focus groups (high and low utilization). Relevant data findings are presented by CFIR constructs: characteristics of individuals, intervention characteristics, inner setting, and outer setting.

#### Characteristics of individuals

**Fig. 2:**
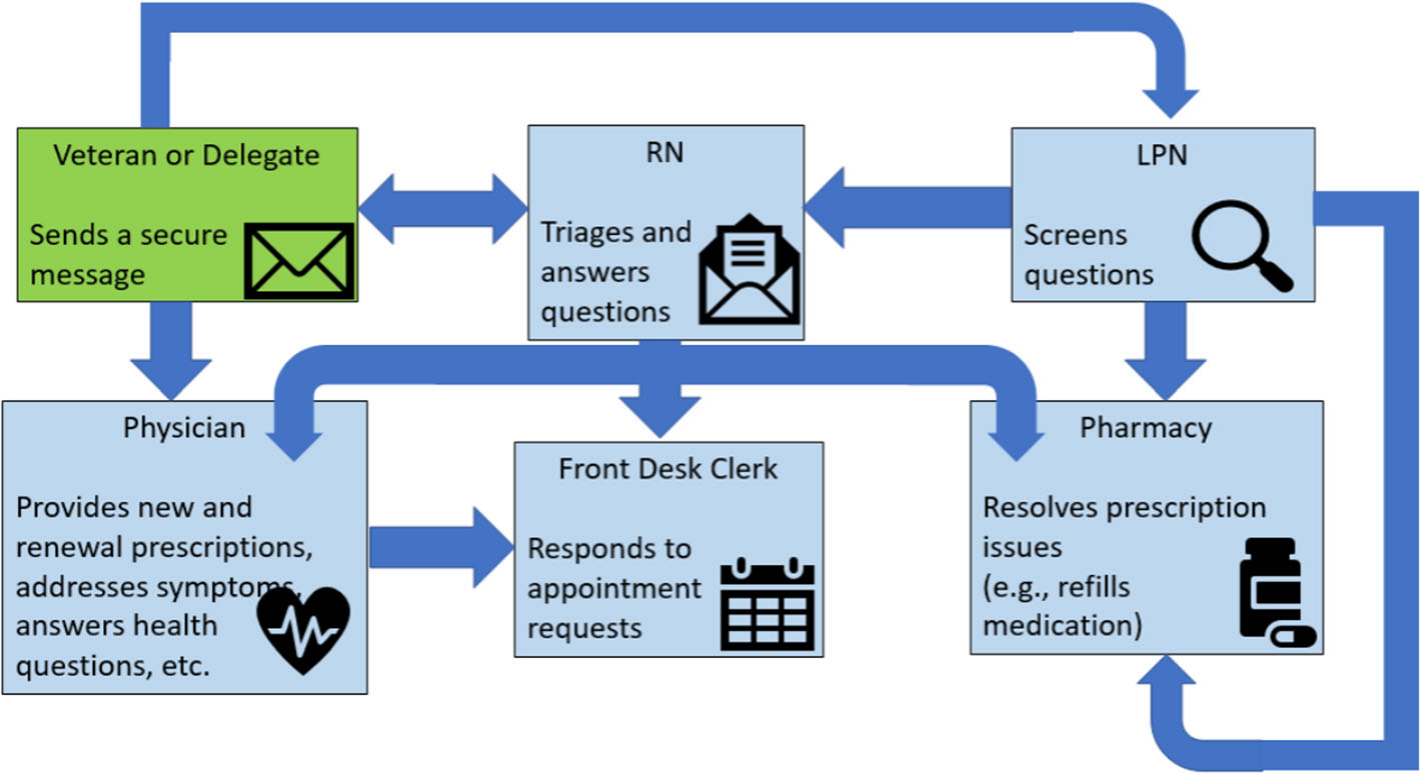
PACT coordinated use of Secure Messaging. RN = Registered Nurse. LPN = Licensed Practical Nurse

*Knowledge and Beliefs about the Intervention* was a factor associated with VHR use. Participants identified using Secure Messaging, telehealth and VetLink Kiosks for patient care. Comfort using VHR varied across groups. Those comfortable using VHR use among those with comfort and knowledge created ease in their work flow as reported by one nurse, ‘We use things (VHR) and we don’t know we are using them…it’s like breathing’ .Participants did not report knowing how to proactively integrate VHR. Participants were more likely to report using VHR when they believed it benefited their patients, improved patient-provider relationships, and enhanced their clinical workflow. One physician reported, “Secure messaging allows for problem-focused response”, saving his time and improving workflow. Though the project was focused on all available VHR, participants consistently described using Secure Messaging and described coordinated use of Secure Messaging (Fig. 2). Participants reported the need for education about coordinating proactive integration of VHR.

#### Intervention characteristics

Five *intervention characteristics* [14] that impact VHR use were: *relative advantage, adaptability,complexity*, and *design quality and packaging,* and *cost*. *Relative Advantage* was germane to participants, who suggested VHR use provided expedited and improved continuity of care which increased patient satisfaction. One physician reported using VHR made for more efficient work. ‘“(VHR) allows providers to work to the top of their licenses” thus allowing registered nurses, advanced practice registered nurses, and physicians the ability to work to the scope of their license, no longer confined by inefficient processes that limit licensed providers’ actions. *Adaptability* was relevant, participants indicated a need for VHR to be tailored and integrated to support workflow needs, though several focus groups did report efforts to create processes to integrate VHR into workflow. *Complexity* was identified as a system-based issue by participants, such that the user experience can be improved through streamlining VHR for ease of navigation and integration. *Design quality and packaging* was a concern reported among participants. Participants cited usability issues and inability to easily navigate between VHR platforms, suggesting efforts to better package and integrate the suite of available VHR as necessary to support their integrated use. *Cost* of time was a clear challenge for participants in their motivated use of VHR as evidenced by reports of negative impacts on workflow and the continued need for team member and patient education.

#### Inner setting

Data gleaned relevant to inner setting, included *networks and communications*, *compatibility, available resources, access to knowledge and information. Networks and communications* emerged as relevant to participants, resulting in team-based development of processes to adjust to workflow impacts. Participants indicated VHR can both positively - and negatively - affect workflow. Though participants stated workflow could be improved by using multiple tools, their workflow was also congested because these systems are often siloed, cumbersome to navigate (e.g., number of clicks) in and between systems (e.g., electronic health record, telehealth), and sometimes malfunctioned (e.g., VetLink Kiosk). *Compatibility* was relevant, as participants reported VHR use aligned with team member and patient norms and values, however the technology was often incompatible within existing workflows. *Available resources* were noted as an issue, such as the need for dedicated time, training, and education, for team members and patients to support adoption and motivated VHR use. A*ccess to knowledge and information* was needed, particularly about best practices and specific use cases/scenarios that are driven by care tasks and performance measures. Several nurses suggested a ‘hear it, see it, and do it’ approach to sharing VHR knowledge and information.

#### Outer setting

Outer setting constructs relevant to participants’ VHR use were *patients’ needs and resources* and *external policy and incentives.* Specific to *patients’ needs and resources,* participants reported encouraging patients to use patient facing tools, however, they reported patient communication preferences to be age dependent, such that older patients preferred phone calls or walk-in appointments. Most teams identified VHR education as part of their routine patient care. Promoting patient use of VetLink Kiosks and telehealth was rarely coordinated as a team, but instead fell to individual team members. When VHR was not a viable option for a patient or the clinic, teams provided alternatives, such as telephone calls. Some primary care teams reported having limited access to Wi-Fi. These teams tended to rely less on VHR answering patients’ questions at the front desk and during walk-in appointments. *External Policy and Incentives* emerged as a construct of relevance, though low user groups more often recommended system changes such as workload credit and dedicated staff to educate patients. These incentives were identified as a critical need to promote proactive integrated VHR use.

#### Implementation strategies for increasing proactive integrated VHR use

Focus group participants identified several implementation strategies to support adoption and VHR use, for example: patient and provider education on VHR; dedicated on-site technical assistance and additional resources to assist with enrollment, and patient identity authentication; and patient-facing test user accounts for providers who may otherwise have no firsthand knowledge of the patient-side of VHR. There were key differences in user groups’ promotion of implementation strategies (Table 4) [16]. In general, the most useful strategies reported were increasing educational approaches (e.g. hands on), changing record systems to assess implementation and clinical outcomes, centralizing technical assistance, assessing locations for readiness, and identifying barriers and facilitators.

#### Expert informant interviews

Expert informant interviews focused on the identification and review of twenty-three VHR tools that could be used across the healthcare continuum (Fig. 1). The right side of Fig. 1 includes a numbered list of VHR participants reported using at various phases of the healthcare continuum. VHR are further described in Table 5 including whether the VHR is veteran or provider facing. Note some VHR faces both veteran and provider. This numbered list was referenced as experts identified tasks and VHR that could be proactively integrated at each phase of the healthcare continuum (Table 6). The double line in the table delineates additional VHR that are currently available to VA staff. Each of these VHR are designated with an asterisk. It is our hope that this table, though not exhaustive, will provide an inventory of many VHR available. Expert informants suggested proactive integrated use of VHR will be predicated on several important factors: (1) primary care teams must view VHR as therapeutic tools and proactively “prescribe” them accordingly; (2) provider-facing VHR must complement existing documentation requirements and tools; and (3) an integrated system of provider and patient generated health data is critical.

**Table 5:**
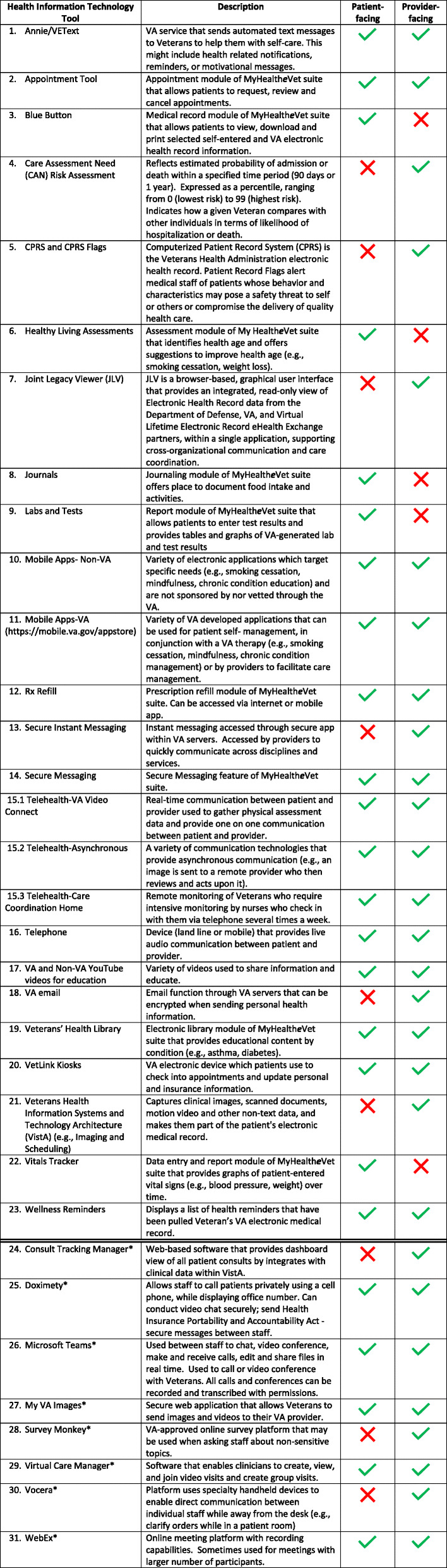
Virtual Healthcare Resources.

* These virtual healthcare resources are currently available to VA staff but were not identified by participants at the time of this study

**Table 6:** VHR use within the healthcare continuum

	Pre-appointment	Check-in/Check-out	Medical Encounter	Post-appointment	Ongoing Health Management
**Veteran Activities**	• Schedule medical visit (2, 14, 16) • List problems to be discussed at appointment (3, 4, 14, 16) • Complete Healthy Living Assessment (6) • Identify symptoms and current treatments (3, 14, 16, 22)	• Review and update personal information (16, 20) • Review and update insurance information (16, 20) • Check-in (20) • Check-out (20) • Schedule follow-up visit (2, 14, 15, 16)	• Measure health indices (9, 15, 22) • Learn about condition (4, 10, 11, 14, 15, 16, 17, 19)	• Seeking information about condition and treatment plan (3, 6, 8, 10, 11, 14, 15,16, 17, 19) • Follow-up with providers (2, 14, 15, 16)	• Self-management activities (1, 6, 8, 10, 11,12, 22, 23) • Monitoring health indices (1, 6, 8, 15, 16, 22) • Managing personal health information (3, 8) • Identifying need for additional medical visits (2, 8, 14, 22, 23) • Communicating with health care team (1, 3, 14, 15, 16) • Labs and test (9)
**Primary Care Team Member Activities**	• Schedule medical visit (14, 15, 16) • Prepare Veteran for appointment (e.g., fast for labs, list concerns to be discussed) (14, 15, 16) • Stratify hospitalization risk (4) • Coordinate with staff in preparation for appointment (5, 13, 16, 18, 21, 23)	• Schedule follow-up visit (14, 15, 16)	• Measure and document health indices (5, 9, 14, 15, 16, 22) • Obtain medical history (3, 5, 7, 14, 15, 16, 21, 22) • Physical examination (15, 16) • Documentation of assessment and plan (5, 21) • Coordinate care with other providers and services (3, 5, 13, 18, 21) • Educate patient on plan and conditions (4, 10, 11, 14, 15, 16, 17, 19, 23) • Prescribe medication (5)	• Send Veteran to Travel for reimbursement on appointment travel (13, 16) • Staff create registered nurse follow-up messages (1, 13, 14, 18) • Home health assessment for personal care needs and follow-up on resources (5, 14, 15, 16)	• Communicating with health care team (5, 13, 14, 15, 16, 21) • Labs and test (5, 9) • Medication reconciliation (5, 12, 14, 15, 16, 17) • Patient data reporting (4, 5, 21, 22) • Educate patient (10, 11, 14, 15, 16, 17, 19, 23)
**Collaborative Activities**	• Discuss Advanced Directives (5, 16) • Prioritize discussion points (14, 15, 16)	• Assist with review and update personal information (16, 20) • Assist with review and update insurance information (16, 20) • Assist with Check-in (20) • Assist with Check-out (20)	• Measure health indices (5, 9, 14, 15, 16, 22) • Discuss concerns/symptoms/current treatment (6, 8, 14, 15, 16) • Labs and tests (5, 9) • Complete required forms (5, 21) • Consenting for procedures (5, 21) • Collect data from journaling, Fitbit, etc. (1, 3, 6, 8, 10, 11, 14, 15, 16, 22)	• Implement treatment plan (1, 5, 8, 10, 11, 19, 22, 23)	• Revise treatment plan (1, 5, 8, 10, 11, 22, 23)

Legend: numbers 1–23 refer to the listed VHR in listed on the right side of Fig. 1

## Discussion

Identifying proactive integrated VHR use best practices is imperative. Proactive integrated VHR use is complex with several factors, including coordination across multiple tools, which can be used across the healthcare continuum. This study aimed to understand how primary care team members used VHR through qualitative and quantitative data. Though teams reported using some VHR, teams did not report using multiple VHR in a proactive integrated way. Use of singular systems was the norm; often use was reactive. Differences and similarities were identified between high- and low- utilization groups in the qualitative findings. Similar to previous publications, all groups described concerns with accessibility, functionality and VHR knowledge [26, 27]. Even though best practices for many primary care tasks and performance measures exist in the literature [28–32], participants from both utilization groups identified the need for VHR best practices that are specific to care tasks (e.g., history taking and consultations) and performance measures (e.g., smoking cessation and chronic pain). These VHR best practices have not been established in the literature [9]. Previous research has shown primary care physician’s familiarity and comfort with the electronic patient-portal influence portal use [33]. These previous findings are in alignment with our findings that teams want to know what VHR patients have access to and how they use it. Due to the pandemic, providing remote care has become a necessity. These findings may inform future implementation efforts.

Data suggest a need to identify and disseminate best practices that can be applied across the healthcare continuum. Interview data presented options for proactively integrating VHR as a standard of care across the healthcare continuum. To our knowledge, proactive integration of VHR across the healthcare continuum does not exist in the literature, making this a novel finding. Table 5 is not an exhaustive inventory, but it is representative of the kinds of information and tools available to primary care teams, needed to engage in proactive integrated VHR use. This information can inform the production of materials and training (i.e., toolkits) to increase awareness, motivate VHR use and initiate a culture change.

The final aim of this study was to identify implementation strategies to promote primary care team use and integration of VHR across the healthcare continuum. Participants identified education and training implementation strategies as a critical need. To our knowledge, there is currently no training content that supports the proactive integrated use of VHR or best practices within the VA. Though focus group participants reported perceiving older patients do not prefer using VHR, previous research has found this to be a misconception [6, 34] For patients who are hesitant to adopt VHR, team members will benefit from efforts to support and reinforce patients’ motivated use and perceived value of VHR [5]. Implementation strategies need to focus on awareness building to dispel this misperception.

A common way to package implementation strategies and best practices is development of a toolkit [35–37]. Though not highlighted in this paper, the parent study used findings from this study aim to develop, and disseminate, a VA primary care team toolkit. This toolkit is currently being finalized and will be accessible via the VA Office of Connected Care.

Finally, consistent with the literature, participants stated their motivation to work on VHR tasks was negatively influenced by time burden [38]. Workload credit and incentives to proactively integrate VHR into the coordination and delivery of care may provide the necessary motivation for adoption and sustained use over time [6, 39].

### Strengths and limitations

This study contributes to the science by providing: (1) current use and potential for proactive integration of VHR into daily workflow; and (2) identification of team focused implementation strategies to increase uptake and proactive integrated use of electronic health resources. The use of CBPR and mixed methods in this study informed primary care team experiences with VHR and incorporated their expertise through the research process. Biases during all phases of the study were limited by integrating participant perspectives, and using mixed methods to triangulate findings [40]. Study team has extensive research experience. BC and CM have worked closely for several years, studying qualitative design and methods. VP has over 5 years’ experience designing innovative data analysis plans, including triangulation. JH worked closely with WM and BZ to conceptualize, design and implement this study.

Limitations should be considered when interpreting findings. First, data represents VHR use at one site, however this site is a large, teaching hospital that treated nearly 100,000 Veterans and conducted over one million outpatient visits at the time of this study. Second, while the sample size was comparable to other qualitative studies [41], based on a representative sample of participants and saturation of data, findings may not be generalizable beyond primary care within comparable healthcare systems. Third, though we purposively recruited primary care members, we may have missed valuable insights from other clinical groups. To address this limitation in part, we expanded recruitment to include primary care pharmacy, nutrition, psychology, and social work. Fourth, though we used CFIR to analyze and contextualize data, it may have biased the lens by which data was interpreted. The CBPR approach and analysis by multiple qualitative experts increases the validity of data collection and findings. Fifth, though utilization groups were sorted by using high and low Secure Messaging as a proxy for VHR use, it is notable they reported no difference in the participant survey – this is likely because the survey queried a dichotomous response of yes/no, not quantity of use. As such, the survey is limited in its usefulness to measure the frequency of Secure Messaging use.

### Future research, education and policy

Findings from this project may inform future research on the use of VHR in specialty care (e.g. rehabilitation, mental health, cardiology). Also, COVID-19 presents as a natural experiment to examine the increased motivation and necessity for proactively integrating VHR into the health care delivery process, research efforts should carefully examine VHR use within the new era of care that emphasizes reducing contact and remote access. Future research should include large scale implementation studies that include clinical and implementation outcomes. As implementation efforts continue, research should focus on the identification and dissemination of best practices. There is continued need for dynamic education to support primary care team proactive integrated VHR use across the healthcare continuum, with emphasis on specific care tasks and performance measures [42]. Education is needed to dispel primary care team member misperceptions about patient preferences for VHR, as well as specialized training to support patient adoption of VHR. Finally, workload credit and incentives should be considered in future research and policy efforts [42].

## Conclusion

Health systems are leveraging technologies to proactively integrate VHR, and maximize information exchange, clinical decision support and patient engagement. Proactive integrated use of VHR is not common practice among primary care teams. Care teams want to know how to proactively integrate VHR into daily workflow, and they want workload credit for incorporating VHR use into healthcare coordination and delivery. Our work to provide trainings, resources and a toolkit helped to improve care team knowledge of proactive integrated use of VHR. Future efforts should build on this toolkit, making VHR and its proactive integrated use common place across primary care teams. Though recent requirements for reducing contact create an intrinsic motivation, cultural change through education and best practices of proactive integrated use across the healthcare continuum is needed to create a culture of VHR super users. Future efforts can work to create culture change and understand proactive integrated use of VHR while maintaining access to comprehensive high-quality care.

## Supplementary Information

**Additional file 1.** PACT Teamlet Member Demographic Survey.

**Additional file 2.** PACT Teamlet Member Virtual Medical Modality Survey.

**Additional file 3.** Aim 1 - PACT Teamlet Member Focus Group Interview Script.

**Additional file 4.** Aim 1 Expert Informant Interview Script.

**Additional file 5.** COREQ (COnsolidated criteria for REporting Qualitative research) Checklist.

## Data Availability

The datasets during and/or analyzed during the current study available from the corresponding author on reasonable request.

## Abbreviations

VHR: virtual healthcare resource
VA: Department of Veterans Affairs
CBPR: community-based participatory research
CFIR: Consolidated Framework for Implementation Research
SD: standard deviation
M: mean
CI: confidence interval
CPRS: Computerized Patient Record System
JLV: Joint Legacy Viewer
VISTA: Veterans Health Information Systems and Technology Architecture
