# Correction to: Proactive integrated virtual healthcare resource use in primary care

**DOI:** 10.1186/s12913-021-06983-3

**Published:** 2021-10-09

**Authors:** Jolie N. Haun, Bridget A. Cotner, Christine Melillo, Vanessa Panaite, William Messina, Shilpa Patel-Teague, Brian Zilka

**Affiliations:** 1 Research and Development Service, James A. Haley VA Hospital and Clinics, 8900 Grand Oak Circle (151R), Tampa, FL 33637-1022 USA; 2 Department of Community & Family Health, College of Public Health, University of South Florida, Tampa, FL USA; 3 Department of Anthropology, University of South Florida, Tampa, FL USA; 4 Department of Psychology, University of South Florida, Tampa, FL USA; 5 James A. Haley Veterans Hospital, Tampa, FL USA; 6 Veterans Integrated Service Network 8 Network Office, St Petersburg, FL USA

**Correction to: BMC Health Serv Res 21, 802 (2021)**

**https://doi.org/10.1186/s12913-021-06783-9**

Following publication of the original article [1], the author figured out several corrections:

1. The title was incorrectly given as ‘Informing Proactive integrated virtual healthcare resource use in primary care’ but should have been ‘Proactive integrated virtual healthcare resource use in primary care’.

2. The affiliation details for author affiliation 1 was incorrectly given as 'Research and Development Service, James A. Haley VA Medical Center, James A. Haley VA Hospital and Clinics, 8900 Grand Oak Circle (151R), Tampa, FL 33637-1022, USA' but should have been 'Research and Development Service, James A. Haley VA Hospital and Clinics, 8900 Grand Oak Circle (151R), Tampa, FL 33637-1022, USA'. ‘James A. Haley VA Medical Center’ is old language that the author’s organization has removed from all communications

3. In Table 2, the third column header should be changed from ^2^ to χ^*2*^.

**Table 2:** Use and promotion of virtual healthcare resources (VHR) among providers from high versus low utilization groups

	LOW (*N* = 29)	HIGH (*n* = 23)	ϰ^***2***^	***p***	***h (95%CI)***
**Provider’s VHR use (% yes)**					
My Health***e***Vet	79.3	73.9	0.21	0.65	0.13 (0.05,0.20)
Secure Messaging	86.2	78.3	0.57	0.45	0.21 (0.13,0.28)
Telehealth	48.3	43.5	0.12	0.73	0.10 (0.02,0.17)
VetLink Kiosks	51.7	69.6	1.70	0.19	0.37 (0.29,0.44)
Mobile Apps	10.3	13.0	0.09	0.76	0.08 (0.01,0.16)
**Patients’ preferred methods of communication (% yes)**					
Telephone	86.2	95.7	1.32	0.25	0.34 (0.27,0.42)
Face to face*	79.3	95.7	2.94	0.09	0.53 (0.45,0.60)
My Health***e***Vet	34.5	43.5	0.44	0.51	0.18 (0.11,0.26)
Secure Messaging*	65.5	87.0	3.14	0.08	0.52 (0.44,0.59)
Telehealth	13.8	8.7	0.33	0.57	0.16 (0.09,0.24)
VetLink Kiosks	20.7	13.0	0.52	0.47	0.21 (0.13,0.28)
Mobile Apps	3.4	8.7	0.65	0.42	0.23 (0.15,0.30)
**Providers’ promotion of patients’ use of VHR (% yes)**					
My Health***e***Vet	86.2	87.0	0.01	0.94	0.02 (−0.05,0.09)
Secure Messaging	93.1	95.7	0.15	0.70	0.11 (0.04,0.18)
Telehealth	48.3	39.1	0.44	0.51	0.19 (0.12,0.26)
VetLink Kiosks	58.6	47.8	0.60	0.44	0.22 (0.15,0.29)
Mobile App	10.3	13.0	0.09	0.76	0.08 (0.01,0.15)
**Promotion of patients’ use of VHR on behalf of providers (% yes)**					
My Health***e***Vet	3.7	5.3	0.21	0.90	0.08 (0.01,0.15)
Secure Messaging	0.0	4.8	3.63	0.16	0.44 (0.37,0.51)
Telehealth	3.6	5.0	0.33	0.95	0.07 (−0.01,0.14)
VetLink Kiosks	16.7	5.3	1.91	0.39	0.38 (0.31,0.45)
Mobile Apps	12.5	5.6	1.37	0.71	0.24 (0.17,0.31)
**% Patients with whom use/promote VHR (% responded 50–100%)**					
My Health***e***Vet	55.6	70.0	1.01	0.31	0.30 (0.23,0.37)
Secure Messaging	63.0	68.4	0.15	0.70	0.11 (0.04,0.18)
Telehealth	20.8	31.3	0.56	0.46	0.24 (0.17,0.31)
VetLink Kiosks	56.5	57.9	0.01	0.93	0.03 (−0.04,0.10)
Mobile Apps	11.8	26.7	1.16	0.28	0.38 (0.31,0.45)

* *p* < .10

4. In the caption of Fig. 2, ‘MD=Medical Doctor and Rx=Prescription’ should be removed, because neither MD nor Rx are listed in the actual figure

**Fig. 2:**
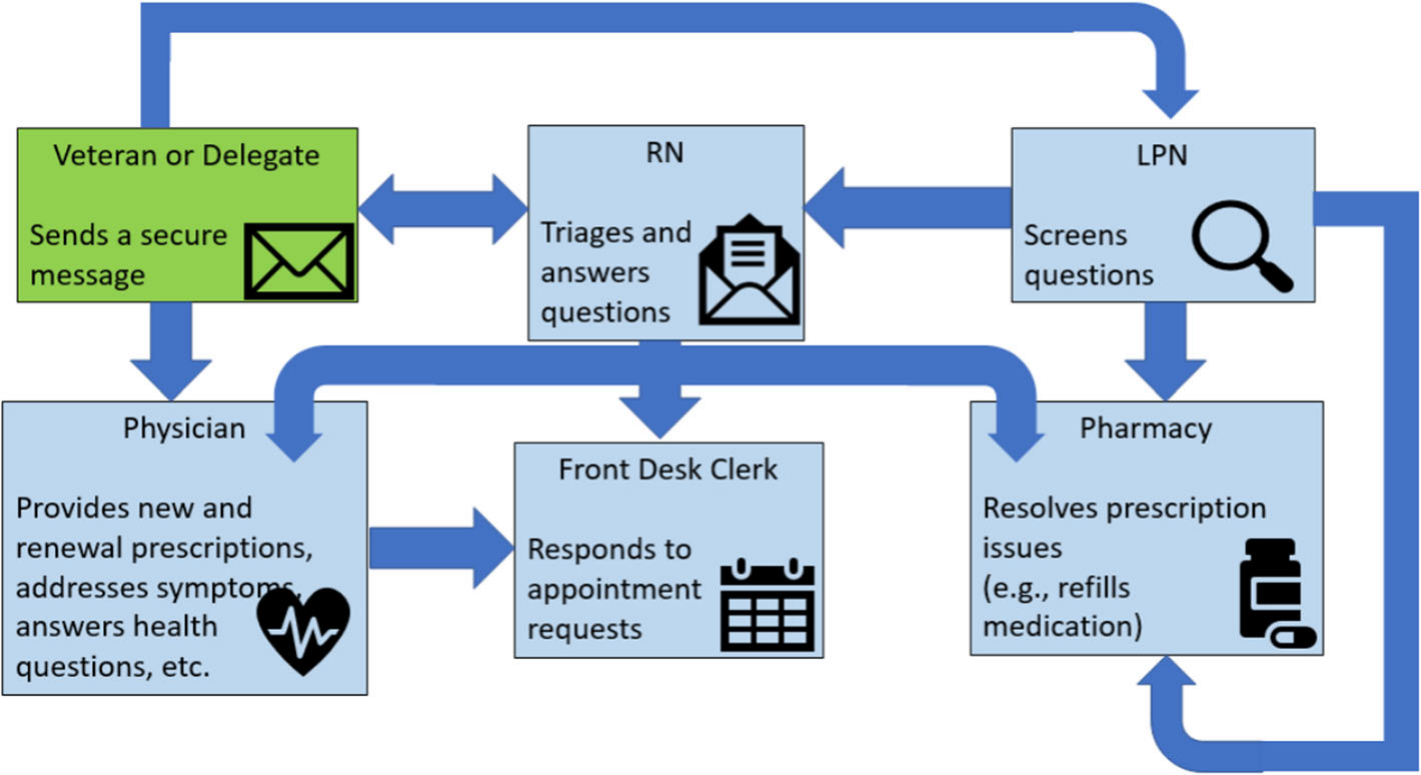
PACT coordinated use of Secure Messaging. RN = Registered Nurse. LPN = Licensed Practical Nurse

The original article [1] has been corrected.
