# Supplementary material for: Proactive integrated virtual healthcare resource use in primary care
Source: BMC Health Serv Res. 2021 Aug 12;21:802. doi: 10.1186/s12913-021-06783-9 (PMC8358911; doi:10.1186/s12913-021-06783-9)
Supplement: Supplementary file 1 — Additional file 1. PACT Teamlet Member Demographic Survey. [file 12913_2021_6783_MOESM1_ESM.docx]

**PACT Teamlet Member Demographic Survey**

**Demographic Items**

| 1. At what facility do you currently work?   Tampa, FL Primary Care Annex  Tampa, FL James A. Haley Main Hospital  Brooksville, FL CBOC  Lakeland, FL CBOC  New Port Richey, FL CBOC  Zephyrhills, FL CBOC |  |
| --- | --- |
| 1. What is your PACT role?   Provider (e.g., MD, DO)  Nurse (e.g., RN)  Clinical Associate (e.g., LPN)  Clerical Associate  Other: ____________________  Does not apply to me   1. How long have you worked at this facility (in years or months)? | yrs. mos. |
| 1. In which unit do you currently work? |  |
| 1. How long have you worked on this unit (in years or months)? | yrs. mos. |
| 1. How long have you worked in the VHA (in years or months)? | yrs. mos. |
| 1. How long have you worked in health care (in years or months)? | yrs. mos. |
| 1. What is your age (in years)? | yrs. |

1. What is your gender?

Female

Male

Other (please specify): _____________

1. Please indicate the race that best describes you. (Check all that apply)

Black, African American

Asian (Chinese, Filipino, Japanese, Korean etc.)

White, Caucasian

Native Hawaiian or other Pacific Islander

American Indian or Alaskan Native

Unknown

Other, please specify ______________________

Decline to respond

1. What is your ethnicity?

Hispanic or Latino

Not Hispanic or Latino

Decline to respond

1. Please provide your highest professional degree. __________________________
2. Please list all your professional licenses.
   1. __________________________
   2. __________________________
   3. __________________________
   4. __________________________
   5. __________________________

**Computer and Internet Use**

1. How often do you use a computer during a typical work day?

≤1 hour a day

1-3 hours a day

4-7 hours a day

≥8 hours a day

Never

Does not apply

1. How often do you use the internet during a typical work day?

≤1 hour a day

1-3 hours a day

4-7 hours a day

≥8 hours a day

Never

Does not apply

1. Are you registered to use MyHealtheVet?

Yes

No

I Don’t Know
