# Supplementary material for: Proactive integrated virtual healthcare resource use in primary care
Source: BMC Health Serv Res. 2021 Aug 12;21:802. doi: 10.1186/s12913-021-06783-9 (PMC8358911; doi:10.1186/s12913-021-06783-9)
Supplement: Supplementary file 2 — Additional file 2. PACT Teamlet Member Virtual Medical Modality Survey. [file 12913_2021_6783_MOESM2_ESM.pdf]

PACT Teamlet Member Virtual Medical Modality Survey

(Sub-Scale 1) Virtual Medical Modality Tool Use Count

1. As a PACT member**,** which of the following virtual care tools have you used with patients? **(Check all that apply)**
   - ☐ Rx Refill (New Rx, Refill, Renewal)
   - ☐ VA wellness reminders
   - ☐ VA appointments and email reminders
   - ☐ VA test results
   - ☐ Blue Button
   - ☐ Secure Messaging
   - ☐ VA allergies and adverse reactions
   - ☐ HealtheLiving Assessments
   - ☐ Veterans Health Library
   - ☐ Telehealth
   - ☐ VetLink Kiosks
   - ☐ Mobile Applications
   - ☐ Other: ________________________________
   - ☐ Does not apply to me

2. If applicable, please list the mobile applications you have recommended to patients.

   ______________________________ ______________________________

   ______________________________ ______________________________

   ______________________________ ______________________________

   ______________________________ ______________________________

(Sub-Scale 2) Virtual Medical Modality Tool Use

3. As a PACT member, how long have you been using virtual care tools (see question 1 for examples)?
   - ☐ Less than six months
   - ☐ Six months to a year
   - ☐ 1-2 years
   - ☐ 3-5 years
   - ☐ More than 5 years
   - ☐ Does not apply to me

4. As a PACT member, how often do you use the virtual care tools (see question 1 for examples) to provide care?
   - ☐ Everyday
   - ☐ Few times a week
   - ☐ Once a week
   - ☐ Few times a month
   - ☐ Once a month
   - ☐ Few times a year
   - ☐ Never
   - ☐ Does not apply to me

(Sub-Scale 3) Patient Virtual Medical Modality Tool Use Count

5. In general, what are your patients' preferred methods of communication and information exchange? **(Check all that apply)**
   - ☐ Telephone
   - ☐ Face-to-face
   - ☐ My HealtheVet
   - ☐ Secure Messaging
   - ☐ Telehealth
   - ☐ VetLink Kiosks
   - ☐ Mobile Applications

6. I promote patients' use of the following virtual care tools. **(Check all that apply)**
   - ☐ Rx Refill (New Rx, Refill, Renewal)
   - ☐ VA wellness reminders
   - ☐ VA appointments and email reminders
   - ☐ VA test results
   - ☐ Blue Button
   - ☐ Secure Messaging
   - ☐ VA allergies and adverse reactions
   - ☐ HealtheLiving Assessments
   - ☐ Veterans Health Library
   - ☐ Telehealth
   - ☐ VetLink Kiosks
   - ☐ Mobile Applications
   - ☐ Other: ________________________________
   - ☐ Does not apply to me

Virtual Care Tool Use

As a PACT member, please answer the following questions about your use of virtual care tools with patients.

7. Have you used or promoted the use of the following virtual care tools with your patients?

	Yes	No, someone else corresponds on my behalf	No	I Don't Know
a. My HealtheVet, including Rx Refill, VA wellness reminders, VA appointments and email reminders, VA test results, Blue Button, VA allergies and adverse reactions, HealtheLiving Assessments, Veterans Health Library (other than Secure Messaging)	☐	☐	☐	☐
b. Secure Messaging	☐	☐	☐	☐
c. Telehealth Services	☐	☐	☐	☐
d. VetLink Kiosks	☐	☐	☐	☐
e. Mobile Applications	☐	☐	☐	☐

8. With what percentage of patients do you use and/or promote the following virtual care tools?

	0%	1%-25%	26%-50%	51%-75%	76%-100%	Does not apply
a. My HealtheVet, including Rx Refill, VA wellness reminders, VA appointments and email reminders, VA test results, Blue Button, VA allergies and adverse reactions, HealtheLiving Assessments, Veterans Health Library (other than Secure Messaging)	☐	☐	☐	☐	☐	☐
b. Secure Messaging	☐	☐	☐	☐	☐	☐
c. Telehealth Services	☐	☐	☐	☐	☐	☐
d. VetLink Kiosks	☐	☐	☐	☐	☐	☐
e. Mobile Applications	☐	☐	☐	☐	☐	☐

As a PACT member, please indicate the extent to which you agree with the following statements.

(Sub-Scale 4) Relative Advantage

9. I think using and/or referring the following virtual care tool(s) with/to patients improves care delivery.

	Strongly Agree	Agree	Neutral	Disagree	Strongly Disagree	Does not apply
a. My HealtheVet, including Rx Refill, VA wellness reminders, VA appointments and email reminders, VA test results, Blue Button, VA allergies and adverse reactions, HealtheLiving Assessments, Veterans Health Library (other than Secure Messaging)	☐	☐	☐	☐	☐	☐
b. Secure Messaging	☐	☐	☐	☐	☐	☐
c. Telehealth Services	☐	☐	☐	☐	☐	☐
d. VetLink Kiosks	☐	☐	☐	☐	☐	☐
e. Mobile Applications	☐	☐	☐	☐	☐	☐

10. I prefer using the following virtual care tool(s) over other traditional tools (face-to-face encounters, phone, postal mail etc.).

	Strongly Agree	Agree	Neutral	Disagree	Strongly Disagree	Does not apply
a. My HealtheVet, including Rx Refill, VA wellness reminders, VA appointments and email reminders, VA test results, Blue Button, VA allergies and adverse reactions, HealtheLiving Assessments, Veterans Health Library (other than Secure Messaging)	☐	☐	☐	☐	☐	☐
b. Secure Messaging	☐	☐	☐	☐	☐	☐
c. Telehealth Services	☐	☐	☐	☐	☐	☐
d. VetLink Kiosks	☐	☐	☐	☐	☐	☐
e. Mobile Applications	☐	☐	☐	☐	☐	☐

(Sub-Scale 5) Observability

11. When PACT members use the following virtual care tool(s) their clinical workflow improves.

	Strongly Agree	Agree	Neutral	Disagree	Strongly Disagree	Does not apply
a. My HealtheVet, including Rx Refill, VA wellness reminders, VA appointments and email reminders, VA test results, Blue Button, VA allergies and adverse reactions, HealtheLiving Assessments, Veterans Health Library (other than Secure Messaging)	☐	☐	☐	☐	☐	☐
b. Secure Messaging	☐	☐	☐	☐	☐	☐
c. Telehealth Services	☐	☐	☐	☐	☐	☐
d. VetLink Kiosks	☐	☐	☐	☐	☐	☐
e. Mobile Applications	☐	☐	☐	☐	☐	☐

12. When PACT members use the following virtual care tool(s) patient outcomes improve.

	Strongly Agree	Agree	Neutral	Disagree	Strongly Disagree	Does not apply
a. My HealtheVet, including Rx Refill, VA wellness reminders, VA appointments and email reminders, VA test results, Blue Button, VA allergies and adverse reactions, HealtheLiving Assessments, Veterans Health Library (other than Secure Messaging)	☐	☐	☐	☐	☐	☐
b. Secure Messaging	☐	☐	☐	☐	☐	☐
c. Telehealth Services	☐	☐	☐	☐	☐	☐
d. VetLink Kiosks	☐	☐	☐	☐	☐	☐
e. Mobile Applications	☐	☐	☐	☐	☐	☐

(Sub-Scale 6) Compatibility

13. I am comfortable using the following virtual care tool(s) to communicate with and deliver care to patients.

	Strongly Agree	Agree	Neutral	Disagree	Strongly Disagree	Does not apply
a. My HealtheVet, including Rx Refill, VA wellness reminders, VA appointments and email reminders, VA test results, Blue Button, VA allergies and adverse reactions, HealtheLiving Assessments, Veterans Health Library (other than Secure Messaging)	☐	☐	☐	☐	☐	☐
b. Secure Messaging	☐	☐	☐	☐	☐	☐
c. Telehealth Services	☐	☐	☐	☐	☐	☐
d. VetLink Kiosks	☐	☐	☐	☐	☐	☐
e. Mobile Applications	☐	☐	☐	☐	☐	☐

(Sub-Scale 7) Complexity

14. PACT members would benefit from education on how to access and the following virtual care tool(s) with patients.

	Strongly Agree	Agree	Neutral	Disagree	Strongly Disagree	Does not apply
a. My HealtheVet, including Rx Refill, VA wellness reminders, VA appointments and email reminders, VA test results, Blue Button, VA allergies and adverse reactions, HealtheLiving Assessments, Veterans Health Library (other than Secure Messaging)	☐	☐	☐	☐	☐	☐
b. Secure Messaging	☐	☐	☐	☐	☐	☐
c. Telehealth Services	☐	☐	☐	☐	☐	☐
d. VetLink Kiosks	☐	☐	☐	☐	☐	☐
e. Mobile Applications	☐	☐	☐	☐	☐	☐

15. It is easy for me to integrate the use of the following virtual care tool(s) into patient care delivery.

	Strongly Agree	Agree	Neutral	Disagree	Strongly Disagree	Does not apply
a. My HealtheVet, including Rx Refill, VA wellness reminders, VA appointments and email reminders, VA test results, Blue Button, VA allergies and adverse reactions, HealtheLiving Assessments, Veterans Health Library (other than Secure Messaging)	☐	☐	☐	☐	☐	☐
b. Secure Messaging	☐	☐	☐	☐	☐	☐
c. Telehealth Services	☐	☐	☐	☐	☐	☐
d. VetLink Kiosks	☐	☐	☐	☐	☐	☐
e. Mobile Applications	☐	☐	☐	☐	☐	☐

(Sub-Scale 8) Context and Facilitation

16. Using virtual care tool(s) at work reflects my PACT role responsibilities.

	Strongly Agree	Agree	Neutral	Disagree	Strongly Disagree	Does not apply
a. My HealtheVet, including Rx Refill, VA wellness reminders, VA appointments and email reminders, VA test results, Blue Button, VA allergies and adverse reactions, HealtheLiving Assessments, Veterans Health Library (other than Secure Messaging)	☐	☐	☐	☐	☐	☐
b. Secure Messaging	☐	☐	☐	☐	☐	☐
c. Telehealth Services	☐	☐	☐	☐	☐	☐
d. VetLink Kiosks	☐	☐	☐	☐	☐	☐
e. Mobile Applications	☐	☐	☐	☐	☐	☐

17. My use of virtual care tool(s) is reinforced in my workplace.

	Strongly Agree	Agree	Neutral	Disagree	Strongly Disagree	Does not apply
a. My HealtheVet, including Rx Refill, VA wellness reminders, VA appointments and email reminders, VA test results, Blue Button, VA allergies and adverse reactions, HealtheLiving Assessments, Veterans Health Library (other than Secure Messaging)	☐	☐	☐	☐	☐	☐
b. Secure Messaging	☐	☐	☐	☐	☐	☐
c. Telehealth Services	☐	☐	☐	☐	☐	☐
d. VetLink Kiosks	☐	☐	☐	☐	☐	☐
e. Mobile Applications	☐	☐	☐	☐	☐	☐

18. I intend to use and/or refer patients to use the following virtual care tool(s) in the future when providing care.

	Strongly Agree	Agree	Neutral	Disagree	Strongly Disagree	Does not apply
a. My HealtheVet, including Rx Refill, VA wellness reminders, VA appointments and email reminders, VA test results, Blue Button, VA allergies and adverse reactions, HealtheLiving Assessments, Veterans Health Library (other than Secure Messaging)	☐	☐	☐	☐	☐	☐
b. Secure Messaging	☐	☐	☐	☐	☐	☐
c. Telehealth Services	☐	☐	☐	☐	☐	☐
d. VetLink Kiosks	☐	☐	☐	☐	☐	☐
e. Mobile Applications	☐	☐	☐	☐	☐	☐
