# Supplementary material for: Proactive integrated virtual healthcare resource use in primary care
Source: BMC Health Serv Res. 2021 Aug 12;21:802. doi: 10.1186/s12913-021-06783-9 (PMC8358911; doi:10.1186/s12913-021-06783-9)
Supplement: Supplementary file 3 — Additional file 3. Aim 1 - PACT Teamlet Member Focus Group Interview Script. [file 12913_2021_6783_MOESM3_ESM.docx]

**Aim 1 - PACT Teamlet Member Focus Group Interview Script**

**Introduction – 8 minutes**

“Thank you for agreeing to participate in this project, an HSR&D funded research study. I will be talking with you today about your experiences using VA virtual care tools, such as My Health**e**Vet features, Telehealth, VetLink Kiosks, and Mobile Applications to coordinate patient care. We will call these tools VMM for short. Specifically, we are interested in your recommendations for increasing PACT team use of these tools.” I’m [name] and I will be facilitating this focus group. [Name} will be taking notes as we go. She may interject from time to time to ask questions or clarify ideas and concepts. You will also notice that you have a blue worksheet in front of you. We will be suing this to complete an activity in just a few minutes.

“I will ask you a series of questions that I would like you to answer openly and to the best of your ability. There is no right or wrong answer. We just want to know about your experiences and what you think about the topic. You can choose not to answer questions or stop participating at any time.”

“We will audio record this discussion. The information you share will be confidential and will not be shared beyond the scope of this project. Remember that any reports from this project that include your responses will not be linked with your name.”

“Do you have any questions?”

*Turn on the recorder, state your name, the date and time, your location and the PACT team you are interviewing*

“To make transcription easier, we would like you to state your first name only and your role on the PACT team. Your name will be stricken from all transcripts. It will take the transcriptionist some time to get to know your voice so be sure you state your name before you speak for the first few questions. Let’s get started!”

**Clinical Experience (RQ.1.1 & PARiHS RQ.1.3):**

**Virtual Medical Modality Use -10 minutes**

1. [*Display list of available virtual care tools*] Which of these tools do you use with your patients? [*Check tools that apply*]
   1. Are there any tools not included on this list that you use with patients?
2. When you use VMM with patients, how do you coordinate that use as a team?
   1. Probe: What are the benefits of working in this way for you and for your patients?

**Awareness of Patient preferences for communication and information exchange-10 minutes**

1. In your experience, what are your patients preferred methods of communication and information exchange?
   - 1. Probe: In your experience, how do patients respond when you promote their use of virtual care tools?
     2. Probe: What alternatives to using virtual tools do you provide patients?

**DOI (RQ.1.2)**

**Relative Advantage:** - 10 minutes

1. How has using virtual care tools affected your clinical workflow?

- 1. Probe: How could these tools be more useful than other traditional care methods?

**Complexity:**

1. What factors make using virtual care tools easy or more difficult to use?

**PARiHS - Context: 5 minutes**

1. What have you done as a PACT team to overcome barriers to using VMM at your site?

**Compatibility:**

1. How could your user experience be changed to better reflect that PACT team’s needs?

**PARiHS (RQ.1.3)**

1. **PARiHS - Facilitation:**

| **ACTIVITY: 30 minutes**  **Observability:**   1. How can VA promote the use of virtual care tools by PACT members?     “We are going to do a brief activity to understand what you feel is needed to promote your PACT team’s use of virtual care tools. At the beginning of the focus group, you were given a blue worksheet. You will use this worksheet for the activity. Let’s go over the worksheet together right now.”  “Each of you has a list of possible strategies for increasing regular use of virtual care tools among PACT team members. Please take a few moments to read this list select ONE strategy that you think would be MOST effective and one strategy that you think would be LEAST effective. On the worksheet you will see three post-it notes. Please write the MOST effective strategy on the green post-it and the LEAST effective strategy on the pink post-it. If there is an effective strategy that you can think of that is not part of the list we have given you, please write that on the yellow post-it. Please take the next five minutes to complete this task.” [Wait 5 minutes]  “Now that you have all written your selections on the post-its, I’m going to ask you to stick your post-its in the corresponding MOST, LEAST, and OPTIONAL locations on the pad at the front of the room.”  [After participants are finished take a moment to read through and summarize the post-its in each category]  [Beginning with the MOST category, ask about each strategy individually]   1. Why do you think that [name strategy] is the MOST effective strategy for promoting or improving PACT teams’ use of virtual care tools? 2. As a group, we would like you to discuss the merits of these strategies to rank order them. [After a few minutes] How did you order these strategies and why? 3. What makes strategies ineffective?   [Finally, in the OPTIONAL category, ask about each strategy individually]   1. Why is it important to [name strategy] in order to promote or improve PACT teams’ use of virtual care tools? |
| --- |
|  |

[End of Interview]

“I don’t have any more questions, unless you have other comments or questions I will turn off the recorder. (Keep recorder on if they have questions or comments – turn off recorder after they state they are finished.)”

“Thanks for your participation!”
