# Supplementary material for: Proactive integrated virtual healthcare resource use in primary care
Source: BMC Health Serv Res. 2021 Aug 12;21:802. doi: 10.1186/s12913-021-06783-9 (PMC8358911; doi:10.1186/s12913-021-06783-9)
Supplement: Supplementary file 4 — Additional file 4. Aim 1 Expert Informant Interview Script. [file 12913_2021_6783_MOESM4_ESM.docx]

**Aim 1 Expert Informant Interview Script**

**Introduction**

“Thank you for agreeing to participate in this expert informant interview. IMy name is… and I will be talking with you today about your experiences using VA virtual care tools, such as My Health**e**Vet features, Telehealth, VetLink Kiosks, and Mobile Applications to coordinate patient care. Specifically, we are interested in finding out more about your role, experiences, perceptions and intentions for using VA virtual care tools. We will refer to these tools as VMM.

“I will ask you a series of questions that I would like you to answer openly and to the best of your ability. There is no right or wrong answer. We just want to know about your experiences and what you think about the topic. You can choose not to answer questions or stop participating at any time.”

“We will audio record this discussion. The information you share will be confidential and and will not be shared beyond the scope of this project. Remember that any reports from this project that include your responses will not be linked with your name.”

“Do you have any questions before we begin?”

1. How do you currently use virtual care tools with your patients to support their health care management tasks?

**PARiHS**

1. What are the primary barriers that PACT teams face when using virtual care tools with patients at your site?
   1. What strategies are used at your site to promote PACT team use of virtual care tools with patients? In your opinion, which of these strategies are most effective?
   2. What are some of the reasons that a strategy may be unsuccessful?
2. We understand that these are some of the strategies for promoting PACT team use of virtual care tools?

[ADD LIST HERE]

- 1. Which of these items do you find useful?
  2. What strategies would you add to this list? Why?
  3. What support structures do you need?

1. What are some key ways you can use virtual care tools at each step of the care delivery process, from pre-appointment, to check-in, to scheduling, to appointment, to follow-up, and self-care management between visits.
2. How would you prefer to be educated about virtual care tools?
3. In summary, we want to know how the VA can promote the use of virtual care tools by PACT members. Is there anything we didn’t discuss that you would like to add?

Thank you so much for taking the time to talk with me; we will use what you have told us today to inform the PACT members’ perspective in our research findings.
